# Intestinal volvulus in the pump twin of a twin reversed arterial perfusion (TRAP) sequence after laser therapy at 18 weeks: a case report

**DOI:** 10.1186/s13256-020-02444-3

**Published:** 2020-08-08

**Authors:** Tania T. Herrera, Katia Rueda, Honorina Espinosa, Gabrielle B. Britton

**Affiliations:** 1 Department of Obstetrics and Gynecology, Pacífica Salud, Panamá, Panamá; 2 Instituto de Investigaciones Científicas y Servicios de Alta Tecnología (INDICASAT AIP), Ciudad del Saber, Panamá, Panamá; 3 Department of Pediatrics, Pacífica Salud, Panamá, Panamá; 4 Division of Pediatrics, Hospital del Niño, Panamá, Panama; 5 Division of Pediatric Surgery, Hospital del Niño, Panamá, Panama

**Keywords:** Intestinal volvulus, Prenatal diagnosis, Twin pregnancy, Fetal therapies, TRAP sequence

## Abstract

**Background:**

Twin reversed arterial perfusion sequence is a rare and potentially lethal condition affecting approximately 1% of monochorionic twin pregnancies and 1 in 35,000 pregnancies overall. An apparently normal (pump) twin perfuses its severely malformed cotwin with deoxygenated blood via retrograde flow in direct arterioarterial anastomoses between the umbilical arteries of each twin. Fetal intestinal volvulus is a rare condition usually manifesting after birth. We report a unique case of twin reversed arterial perfusion sequence in association with intestinal volvulus in the surviving pump twin.

**Case presentation:**

A 32-year-old Hispanic primigravida was referred to our clinic after a fetoscopy procedure of laser photocoagulation of anastomoses at 18 weeks of gestation. Follow up scans in the ex-pump twin revealed dilated bowel loops and a typical “whirlpool sign” at 26 weeks of gestation, and intrauterine intestinal volvulus was suspected. At 29 weeks of gestation, preterm premature rupture of membranes occurred, and an emergency cesarean section was performed. The newborn was diagnosed in the early neonatal period with intestinal perforation. The diagnosis was postnatally confirmed by surgery and histopathology.

**Conclusions:**

The type of fetal intervention and late gestational age of the procedure increase the risk of complications. This case alerts health providers to be vigilant in the follow-up of patients with complicated monochorionic pregnancies.

## Background

**Fig. 1:**
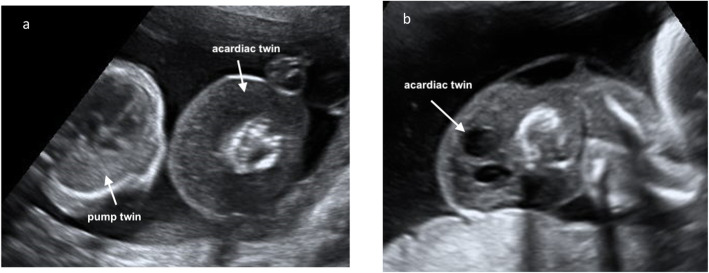
Ultrasound images of a twin reversed arterial perfusion (TRAP) sequence. **a** Transverse view of both the pump and acardiac twin, *white arrows*. **b***White arrow*, longitudinal view of the acardiac twin

Twin reversed arterial perfusion (TRAP) sequence is a rare and potentially lethal condition affecting approximately 1% of monochorionic twin pregnancies and 1 in 35,000 pregnancies overall [1]. An apparently normal (pump) twin perfuses its severely malformed cotwin (TRAP or acardiac mass) with deoxygenated blood via retrograde flow in direct arterioarterial anastomoses between the umbilical arteries of each twin (Fig. 1a, b). The anomalous twin, or acardius, then returns further deoxygenated blood back to the pump twin through a direct venovenous anastomosis. This vascular arrangement predisposes to a hyperdynamic circulation and progressive high-output cardiac failure in the pump twin [1–3]. The phenotype of the acardiac twin is classified according to the mode of development and growth: acardius acephalus (no cranial or thoracic structures), acardius anceps (some cranial structures), acardius amorphous (the most malformed structure), and the rarest, acardius acormus, that exhibits cranial elements but no body structure [4]. Although the pump twin is usually structurally normal, there is approximately a 10% incidence of major malformations [4]. One study found a 25% incidence of fetal skeletal malformations among pump twins [4, 5], and gastroschisis [6] and VACTERL associations (vertebral defects, anal atresia, cardiac defects, tracheoesophageal fistula, renal anomalies, and limb abnormalities) [7] have also been reported. Mortality of the pump fetus increases depending on heart failure and prematurity caused by polyhydramnios. Fetal invasive intervention is intended to relieve the increased hemodynamic load, thus preventing congestive heart failure. Different fetoscopic techniques, such as cord coagulation, cord ligation, and photocoagulation of the anastomoses, as well as intrafetal methods, such as radiofrequency ablation (RFA) and intrafetal laser therapy, have been proposed as a means of preventing the demise of the pump twin [1–3].

Intestinal volvulus is a rare condition in which the small bowel and proximal colon twist around the superior mesenteric artery. This occurs when the intestinal loop that is suspended along the free margin of the mesentery twists around the anterior mesentery artery [8–10]. Between the eighth and tenth weeks of gestation, malrotation of the fetal gut occurs when the elongating intestine returns to the abdominal cavity. Between the sixth and tenth weeks, the midgut loop rotates 90 degrees counterclockwise around the axis of the superior mesenteric artery [8–10]. This brings the duodenal jejunal loop to the right and the ileocolic loop to the left side. During the tenth and 11th weeks, the intestines return to the fetal abdominal cavity. Both the proximal and distal loops undergo a 270-degree rotation. The duodenojejunal and ileocolic loops end up posterior and anterior, respectively, to the superior mesenteric artery [8, 9]. In monochorionic twins, congenital anomalies occur frequently and are generally separated into malformations, disruptions, and deformations [11]. Disruptions in monochorionic twins are probably related to the shared placental circulation, which is associated with vascular connections that lead to secondary disruptions [11].

If the TRAP sequence is left untreated, a substantial proportion of pump twins die *in utero* up to 18 weeks of gestation, and 50% of the remainder die in the further course of pregnancy or in the neonatal period as a result of prematurity [12]. These fetal intervention techniques target the umbilical cord vessels, the intrafetal vessels, or the vascular anastomoses on the placental surface [12, 13]. The optimal treatment technique and timing of intervention in pregnancies complicated with TRAP sequence are still debated [1–3].

## Case presentation

A 32-year-old Hispanic primigravida with a spontaneous pregnancy was referred to our institution after a fetoscopic procedure of laser coagulation of anastomoses at 18 weeks of gestation due to TRAP sequence. She was married and had moved to Panama from Venezuela. She did not have any chronic medical illness and lived in a suburb that was well served. At the time of ultrasound examination, the fetal anatomy was normal, and amniocentesis revealed a normal male karyotype (46,XY). At 22 weeks of gestation, symptoms of premature labor emerged, including uterine contractions and effacement of the cervix. Nifedipine was used as a tocolytic medication. Follow-up scans were obtained in search of progressive polyhydramnios and increasing dimensions in the acardiac twin, and signs of cardiac insufficiency were evaluated, including increased cardiac dimensions, tricuspid valve regurgitation, and pericardial effusion in the ex-pump twin. The result of fetal echocardiography performed at 25 weeks of gestation was normal. See Table 1.

**Table 1:** Timing of events during prenatal consults

Dates	Relevant past medical history and interventions		
21 May /2013	18 + 6 weeks: Laser photocoagulation of arterioarterial and venovenous anastomosis, 3.2-mm endoscope	TRAP/pump ratio, 120% VPM, 10 cm	
	Summaries from initial visit and follow-up	Diagnostic test	Interventions
10 June 2013	Presented to the clinic after a fetoscopy procedure: Arterioarterial anastomoses for follow-up and management	Ultrasound scan	
		TRAP/pump ratio, 100%	
13 June 2013	22 weeks: Clinical contractions, tocolytic therapy	Cervical length, 3.9 cm	Sample sent to laboratory ruled out infection
		MVP, 6.2 cm	
12 July 2013	26 weeks: Fetal echocardiography by pediatric cardiologist was reported normal	Ultrasound scan	
		Striking tubular dilation of intestines	
		Cervical length, 3.8 cm	
7 August 2013	29 + 6 weeks: PPROM	Magnesium sulfate for 24 hours	
		Betamethasone 12 mg intramuscularly, two doses	
10 August 2013		Male with no other anomalies Weight, 1458 g	Cesarean section
11 August 2013	Neonatal examination	Postnatal image of pneumoperitoneum	Primary ileostomy

*MVP* maximal vertical pocket, *PPROM* Preterm premature rupture of membranes, *TRAP* Twin reversed arterial perfusion

**Fig. 2:**
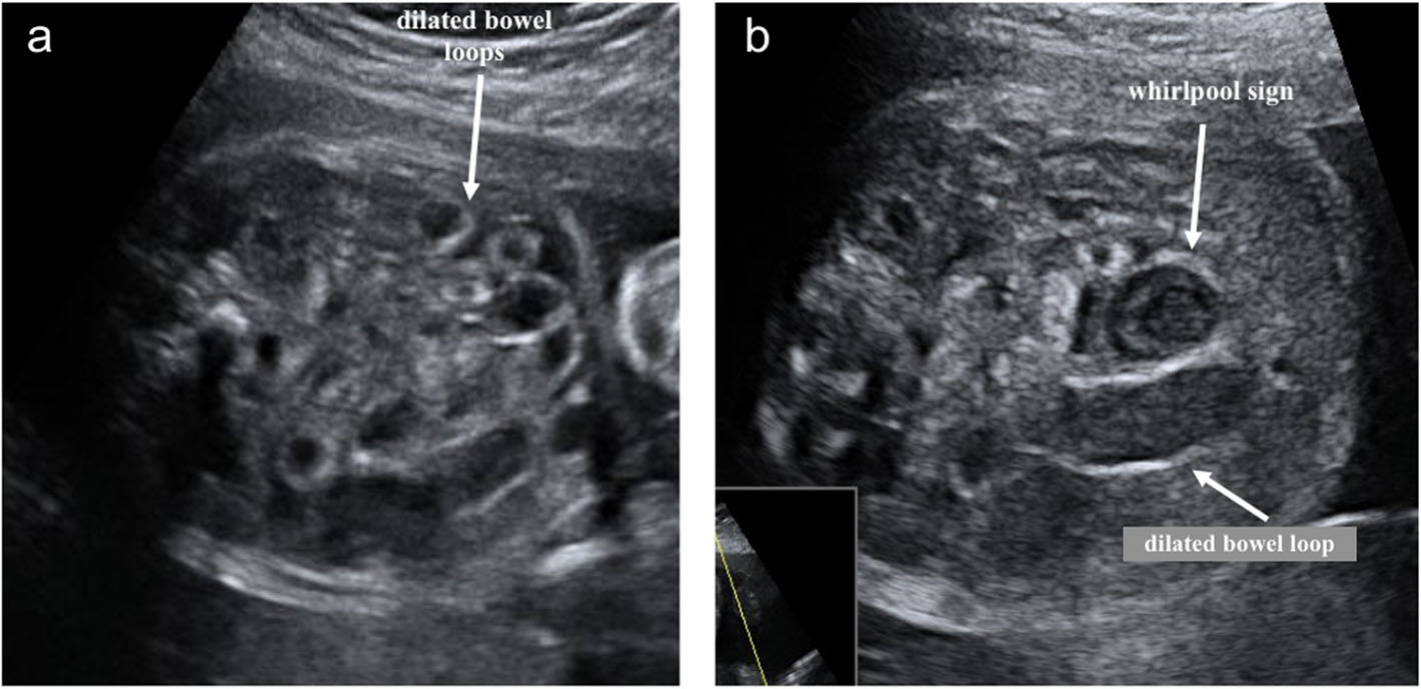
Ultrasound image of fetal volvulus in pump twin. Transverse view of the fetal abdomen with *white arrows* pointing to the (**a**) dilated bowel loop segments and (**b**) dilated bowel loop in a whirlpool configuration

**Fig. 3:**
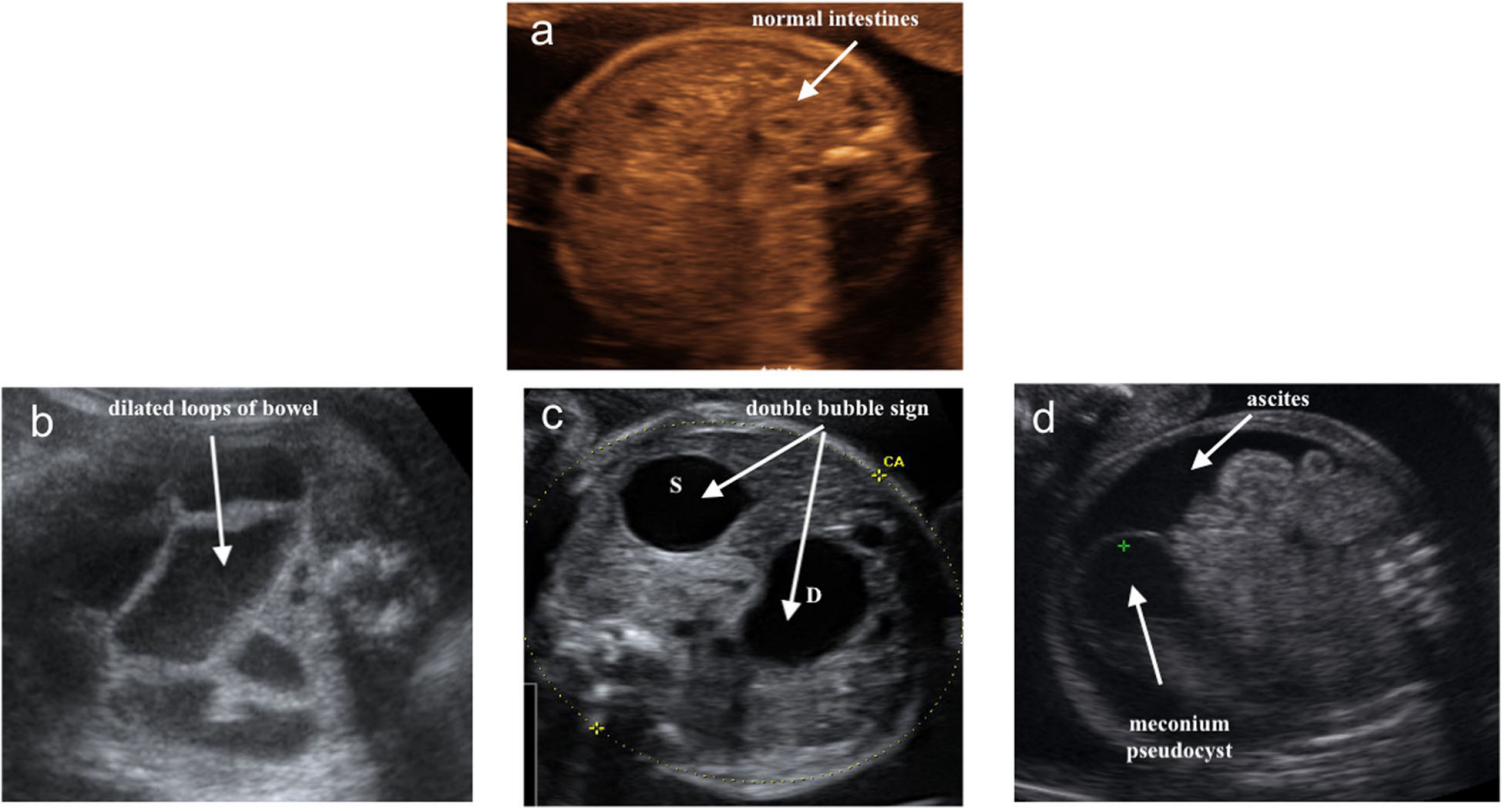
Ultrasound images comparing normal intestines and different signs of fetal bowel obstruction. **a** Normal intestines at 26 weeks of gestation, *white arrows*. **b** Ileal atresia in a fetus with gastroschisis at 30 weeks of gestation. **c** Double bubble sign (*white arrows*; S, stomach; D, duodenum) in a fetus with duodenal atresia at 28 weeks of gestation. **d***White arrows*, ascites and meconium pseudocyst at 22 weeks of gestation

At 26 weeks of gestation, ultrasound scans showed a striking tubular dilation of the lower digestive tract (Fig. 2a, b). On color or power Doppler examination, there was no identifiable flow signal within or between the dilated bowel loops. At that time, the differential diagnoses were jejunal atresia, ileal atresia, meconium ileus, intestinal volvulus, and meconium peritonitis (Fig. 3). Umbilical artery Doppler was normal. At 29 + 6 weeks, after preterm premature rupture of membranes (PPROM), magnesium sulfate for 24 hours and an antenatal corticosteroid (betamethasone) was administered. A male infant weighing 1458 g was born via cesarean section. The result of neonatal examination of the abdomen was normal. The newborn was diagnosed in the early neonatal period with intestinal perforation. Exploratory laparotomy confirmed volvulus and intestinal malrotation, and end-to-end anastomosis was performed. The midportion of the small bowel contained a closed bowel obstruction that was strangled and became gangrenous. The volvulus was untwisted, and the gangrenous segment with a sealed perforation was resected. A primary ileostomy was performed with resection of 10 cm of distended ileum. The newborn’s postoperative course was uneventful, and intestinal continuity was restored. Oral feeds began 10 days after the procedure, with the patient previously receiving total parenteral nutrition. The boy is now 5 years old, and his clinical assessments are normal. Written informed consent was obtained for the publication of the present case report.

## Discussion

To our knowledge, this is the first case report of prenatally diagnosed intestinal volvulus in TRAP sequence after fetoscopy treatment. This case alerts health providers to be vigilant in the follow-up of patients with complicated monochorionic pregnancies.

Although the pump twin is usually structurally normal, there is an approximately 10% incidence of major malformations [4]. In a series of 12 TRAP cases, 3 (25%) were noted to have fetal skeletal malformations. One of these had a lethal skeletal dysplasia, another had abnormal fourth and fifth digits of the upper extremities, and the last one had hemivertebrae [4, 5]. Another case report described an upper limb reduction defect [5]. Gastroschisis [6], VACTERL association [7], and transposition of great arteries have been reported in the pump twin [11]. In our patient’s case, no skeletal defects or congenital heart defects were found.

Intestinal volvulus is a rare condition in which intestinal loops are twisted around the axis of the pedicle of the superior mesenteric artery. Volvulus commonly occurs in neonates and young infants and infrequently in later life. It is a surgical emergency associated with high morbidity and mortality, and it can occur during intrauterine life with high rates of complications [8, 14, 15]. Most cases of volvulus in infants are associated with intestinal malrotation. Other causes have also been reported, including cystic fibrosis, gastrointestinal duplication, and the presence of either a cyst or a tumor mass [16].

It is a surgical emergency in the postnatal period, with the child’s survival and functional prognosis dependent on the delay until treatment [16]. Timing and place of delivery must be planned to offer appropriate treatment as soon as possible after birth [14].

Sonographic prenatal diagnosis of intestinal volvulus is rare and often difficult in the absence of specific signs. The coffee bean sign and the whirlpool sign and other sonographic patterns, including polyhydramnios, ascites, dilated bowel loops, peritoneal calcifications, and meconium pseudocysts, have been described [14–16] (Table 2). A frequent complication is intestinal ischemic necrosis with consequent bowel perforation [16]. In our patient’s case, the whirlpool sign was first seen around 26 weeks of gestation, almost 7 weeks prior to the average gestational age of presentation of the sonographic signs in larger series (32.5 weeks ± 2.6 weeks) [14–16]. Most recently, in the largest retrospective series to date, authors reported that prenatal volvulus is more often diagnosed at two peaks of gestational age at 27 weeks and 32 weeks, sometimes induced by maternal perception of a decrease of fetal movements [16].

**Table 2:** Diagnostic signs of intestinal volvulus

Bowel loop dilation	
Fluid meconial level	
Whirlpool sign or snail sign: dilated bowel loops forming a typical convoluted mass in a clockwise direction	
Meconium peritonitis: calcification, ascites, pseudocyst	
Coffee bean sign: distension of a very short segment of bowel	

The diagnosis of intestinal volvulus does not necessarily require immediate intervention. Serial fetal monitoring at 28 weeks of gestation and sonographic follow-up in 2 weeks should be performed. If the scans show progressive stabilization of the bowel loop dilation and fetal movements are normal, an observational approach could be established. If the scans show ascites, absence of intestinal peristalsis, decreased fetal movements, and sudden changes in dilation and reduction of intestinal bowel, urgent delivery is advised [14–16].

In the TRAP sequence, it is considered that arterioarterial and venovenous anastomoses between both twins lead to retrograde blood flow from a pump twin to an acardiac twin. Various treatments to improve the outcome of the pump twin have been reported, but the optimal method and timing of treatment are yet to be established [17]. Interventions such as extrafetal methods, including cord ligation, cord coagulation laser, and monopolar or bipolar cord coagulation, and intrafetal methods, such as alcohol, laser RFA, and monopolar coagulation, have been proposed [12].

PPROM, which leads to preterm labor, is a common complication after laser photocoagulation of anastomoses. Chorioamniotic membrane separation, an iatrogenic complication, occurs in approximately 20% of patients with laser photocoagulation [17, 18]. Maternal complications have also been reported in 10.7% of 150 laser photocoagulation cases, with 6.0% classified as major and 4.7% as mild. Major maternal complications included placental abruption, accounting for the majority, as well as amniotic fluid embolism and mirror syndrome [18].

## Conclusion

Malformations in the pump twin, although rare, can happen and should be discussed with the patients. This case alerts health providers to be vigilant in the follow-up of patients with complicated monochorionic pregnancies.

## Data Availability

Not applicable.

## Abbreviations

PPROM: Preterm premature rupture of membranes
RFA: Radiofrequency ablation
TRAP: Twin reversed arterial perfusion
VACTERL: Vertebral defects, anal atresia, cardiac defects, tracheoesophageal fistula, renal anomalies, and limb abnormalities
